# Vertical Hydrodynamic Focusing and Continuous Acoustofluidic Separation of Particles via Upward Migration

**DOI:** 10.1002/advs.201700285

**Published:** 2017-12-22

**Authors:** Husnain Ahmed, Ghulam Destgeer, Jinsoo Park, Jin Ho Jung, Hyung Jin Sung

**Affiliations:** ^1^ Department of Mechanical Engineering KAIST Daejeon 34141 South Korea

**Keywords:** acoustic radiation force, acoustofluidics, particle separation, travelling surface acoustic waves, vertical components

## Abstract

A particle suspended in a fluid within a microfluidic channel experiences a direct acoustic radiation force (ARF) when traveling surface acoustic waves (TSAWs) couple with the fluid at the Rayleigh angle, thus producing two components of the ARF. Most SAW‐based microfluidic devices rely on the horizontal component of the ARF to migrate prefocused particles laterally across a microchannel width. Although the magnitude of the vertical component of the ARF is more than twice the magnitude of the horizontal component, it is long ignored due to polydimethylsiloxane (PDMS) microchannel fabrication limitations and difficulties in particle focusing along the vertical direction. In the present work, a single‐layered PDMS microfluidic chip is devised for hydrodynamically focusing particles in the vertical plane while explicitly taking advantage of the horizontal ARF component to slow down the selected particles and the stronger vertical ARF component to push the particles in the upward direction to realize continuous particle separation. The proposed particle separation device offers high‐throughput operation with purity >97% and recovery rate >99%. It is simple in its fabrication and versatile due to the single‐layered microchannel design, combined with vertical hydrodynamic focusing and the use of both the horizontal and vertical components of the ARF.

## 1 Introduction

Advancements in microfluidic separation devices are vital for the development of future lab‐on‐a‐chip technologies with applications in the biological, chemical, and materials sciences. Over the past few decades, microfluidic particle and cell separation techniques have played a vital role in cell biology, disease diagnostics, drug screening, drug discovery, and biochemical analysis.^1^, ^2^ To date, many researchers have explored various microfluidic methods for manipulating particles inside minute volumes of fluids, such as inertial microfluidics,^3^ hydrodynamic filtration,^4^ magnetophoresis,^5^ dielectrophoresis,^6^, ^7^ optofluidics,^8^, ^9^ and acoustophoresis.^10^, ^11^, ^12^, ^13^, ^14^, ^15^, ^16^ Acoustophoresis‐based microfluidic separation techniques are preferred due to the contactless handling of the biological samples, low power requirement, and biocompatible nature of the acoustic waves, all of which permit incorporation of acoustophoresis techniques into microscale total analysis systems. Surface acoustic wave (SAW)‐based particle separation devices mostly feature a single‐layered polydimethylsiloxane (PDMS) microfluidic channel and a pair of interdigitated electrodes patterned onto a piezoelectric substrate that produces acoustic waves along the surface of the substrate to manipulate the suspended micro‐objects.^17^, ^18^, ^19^, ^20^

SAWs are classified into standing surface acoustic waves (SSAWs) and travelling surface acoustic waves (TSAWs).^21^ A SSAW is a combination of two constructively interfering TSAWs propagating in opposite directions. SSAWs form pressure nodes and antinodes inside the fluid and are used to push particles toward regions of low pressure inside the microchannel, achieving particle focusing and separation.^22^, ^23^, ^24^ On the other hand, TSAWs have been used in cross‐type acoustic particle separators to laterally migrate particles and realize separation across the microchannel width or within a sessile droplet, because particles predominantly migrate within the horizontal plane.^11^, ^25^, ^26^, ^27^ Most SAW‐based acoustofluidic separation techniques utilize forces that act on micro‐objects suspended in a horizontal plane while pushing them laterally inside the microchannel.^28^, ^29^, ^30^, ^31^, ^32^ The interaction between TSAWs and the fluid results in leaky acoustic waves that radiate at an angle of ≈22° (in systems comprising water and a lithium niobate (LiNbO_3_) substrate) inside the microfluidic channel, such that the vertical component (*F*_v_) of the acoustic radiation force (ARF) acting on the suspended particles is ≈2.5 times greater than the horizontal component of the force (*F*_h_), i.e., *F*_v_ ≅ 2.5 *F*_h_.^33^ The SAW‐based acoustofluidic devices that utilize the horizontal component of an ARF are composed of an interdigitated transducer (IDT) integrated into the side of a single‐layered PDMS microchannel.^30^, ^34^, ^35^, ^36^ (see Figure S1a in the Supporting Information). A multiple‐layered PDMS microchannel with a post beneath the micro‐object manipulation zone was also used in a similar fashion to deflect particles^30^ and sort cells or droplets.^28^ IDTs may also be positioned directly beneath a microchannel to induce desired particle migration. Collins et al. used a single IDT to produce standing acoustic waves within a single‐layered microchannel to achieve particle separation.^37^ However, TSAWs, produced by an IDT placed beneath a single‐layered microchannel, have not yet been demonstrated to be capable of inducing vertical particle migration by imposing a direct ARF onto particles directly from the bottom of the microchannel (see Figure S1b in the Supporting Information). Previously, our research group developed a cross‐type particle separation device that showed vertical particle migration, although the effect was not harnessed for an application.^38^ Collins et al.^33^ took advantage of the vertical component of the ARF and employed a focused IDT to trap and concentrate selected particles behind a microfabricated PDMS membrane. However, the particle trapping capacity of the membrane would reach a saturation value in a short period of time; therefore, a continuous flow separation device offers improved particle separation performance.^20^, ^23^

In the present work, a straight IDT was deployed with a straight loosely aligned microchannel positioned perpendicularly to the uniformly spaced metal electrodes. Compared with the most SSAW‐based devices^23^, ^39^, ^40^ that usually require a pair of parallel IDTs tightly aligned with the microchannel, the present device did not require tight alignment with the microchannel similar to other TSAW‐based devices.^20^, ^38^ However, tilted angle SSAWs have been utilized to circumvent such limitations.^41^ The proposed design provided an additional advantage of utilizing both the horizontal and stronger vertical components of the ARF acting on the particles. The height and width of the microchannel did not significantly alter the device performance, unlike the SSAW‐based devices, in which the microchannel aspect ratio significantly affected the locations of the pressure nodes and antinodes.^22^, ^39^, ^40^, ^42^ The present device utilized a comparatively low input power because the energy loss to PDMS walls was minimal as the IDT was directly exposed to the fluid and both the vertical and horizontal components of the ARF were employed. A single‐layered PDMS microchannel with a simple design was used to continuously isolate the selected particles based on their diameters by inducing vertical migration. Furthermore, particle focusing based on two sheath flows, as used in most SAW‐based particle separation devices,^23^, ^31^, ^43^, ^44^ was circumvented in the present work by focusing the particles along the vertical direction using a single sheath flow. Note that some splitt fractionation or H‐filter‐based separation devices used a single sheath flow configuration. For instance, Hawkes et al.^45^ used a single sheath flow to focus the yeast cells along the side of the microchannel wall prior to washing them using SSAWs. However, the migration of the cells was in horizontal direction and the device required a fabrication of multiple‐layered microchannel. Recently Chen et al.^46^ demonstrated the separation of platelets from whole blood by focusing the biological sample vertically and then pushing them upward using the bulk acoustic waves (BAWs) in a multiple‐layered microchannel. In contrast, the present device strictly distinguishes the external force components (horizontal from vertical), which is available with TSAW device only. The mechanism for SSAW‐and BAW‐based devices would be different. Among the single‐layered PDMS‐based devices, similar kind of focusing is not reported before. Due to the utilization of the principal component of ARF, the present device successfully operated for particle separation at net flow rate up to 1.3 mL min^−1^, which is ≈100 times higher than used in previously reported SAW devices.^20^, ^23^, ^31^, ^32^, ^37^, ^38^, ^42^, ^43^, ^47^, ^48^ The throughput of the proposed device can be further improved with ease by increasing the width of the microchannel since the microchannel is placed directly on the IDT. This low‐power, high‐throughput tiny device will be useful for point‐of‐care testing applications based on biological sample separation.

## 2 Working Mechanism

**Figure 1:**
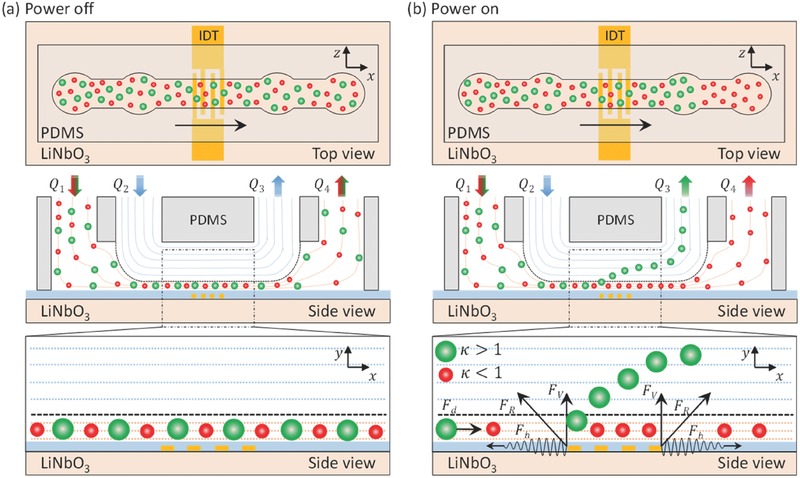
A schematic illustration showing the vertical migration of particles to realize size‐based separation. The separation device is composed of a straight IDT patterned on the lithium niobate (LiNbO_3_) substrate, a SiO_2_ layer, and a straight PDMS microchannel mounted on the top. The top and side views of the device are illustrated as the particles separation zone is enhanced when the power was turned a) off and b) on, respectively. Particles are slowed down by the horizontal component of the ARF *F*_h_ and pushed in the upward direction depending on particle sizes due to the vertical component of ARF *F*_v_.

A schematic diagram of the particle separation device displays a simple PDMS microchannel attached to a piezoelectric substrate (LiNbO_3_) with an IDT deposited on the top of it (see **Figure** **1**). A straight PDMS microfluidic channel consisting of two inlet ports and two outlet ports was mounted on the top of the IDT in such a way that the IDT was positioned between the second inlet and the first outlet. A mixture of two different‐sized particles (larger green and smaller red) was injected through the first of the two inlets at a flow rate of *Q*_1_, and a sheath fluid in the form of deionized (DI) water was introduced through the second inlet at a flow rate of *Q*_2_. The purpose of the sheath flow was to pinch the sample mixture in the lower streamlines using the DI water flowing in the upper streamlines to create a vertical double‐layered flow while hydrodynamically focusing the particles close to the bottom of the microchannel.^49^, ^50^ The first of the two outlets was used to pump out the fluid at a flow rate of *Q*_3_ by applying a negative pressure, while the remaining fluid was collected through the second outlet, which was open to the atmosphere, with a flow rate of *Q*_4_. The sheath flow induced all particles to flow through the lower fluidic streamlines, and particles could be collected through the second outlet, without separation when the power was off (see Figure 1a). Previous studies demonstrated that the value of the κ‐factor, defined as κ  =  *πdf*/*c*_f_, where *d* is the particle diameter, *f* is the TSAW frequency, and *c*_f_ is the speed of sound in the fluid, could be used to effectively characterize the particle motion under a TSAW.^21^ The TSAW frequency and the particle diameters were chosen such that κ > 1 for larger (green) particles and κ < 1 for smaller (red) particles. Once the AC signal was applied to the IDT, it generated an acoustic wave that applied a significant ARF to the larger (green) particles in the horizontal and vertical directions. The horizontal (*X*‐direction) component of the ARF (*F*_h_) slowed down the particles against the direction of the flow due to the resultant drag force *F*_d_ on the particle, whereas the major (*Y*‐direction) component of the ARF *(F*_v_ *)* pushed the green particles vertically upward into the upper streamlines, which were ultimately collected through the first outlet. On the other hand, smaller (red) particles continued to flow in the lower streamlines, nearly unaffected by the ARF, and were ultimately collected through the second outlet (see Figure 1b). As a result, the green particles and red particles were continuously separated through the first and second outlets based on their size difference.

**Figure 2:**
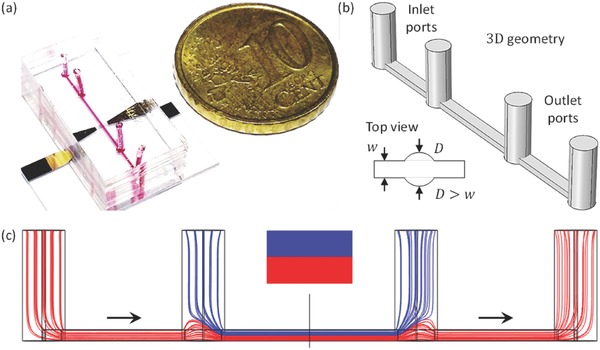
a) A fabricated particle separation device. The PDMS microchannel was bonded on the top of the gold electrodes deposited onto a LiNbO_3_ substrate. Red dye was used to highlight the microchannel and its ports. b) The 3D solid geometry of the microchannel, with a top view. c) Side view of simulated streamlines within the straight microchannel.

For size comparison, the fabricated particle separation device was placed next to a Euro 10 cent coin, as shown in **Figure** **2**a. A red dye was used to highlight the microchannel along with the inlet and outlet ports. The vertical migration of particles induced by the stronger *Y*‐directional component of the ARF could be harnessed to induce particle separation only in the presence of the double‐layered flow within the microchannel. The streamlines in the microchannel were simulated using various inlet and outlet port sizes to optimize the geometry for stabilizing the double‐layer flow. The 3D geometry of the microchannel is shown in Figure 2b. If the diameters of the inlet and outlet holes were smaller than the width of the microchannel, most of the streamlines from the first inlet followed a path around the streamlines coming from the second inlet. This flow resulted in a horizontally triple‐layered flow that was not suitable for separating particles, because they were not adequately focused prior to exposure to the TSAW (see Figure S2a in the Supporting Information). If the size of the punched holes was equal to (see Figure S2b in the Supporting Information) or larger than the width of the microchannel, however, the device favored the desired double‐layered flow, as the streamlines (blue) emerging from the second inlet readily pinched the streamlines (red) emerging from the first inlet (see Figure 2c). The streamlines originating from the first and second inlets were collected through the second outlet and first outlet, respectively. In general, it was not possible to manually punch inlet/outlet holes with a diameter equal to the width of the microchannel; therefore, a microchannel design with inlet and outlet ports larger than the microchannel width was preferred. A top view of the microchannel inlet port, shown in Figure 2b, reveals that the diameter *D* of the punched hole exceeded the width *w* of the microchannel to form a double‐decker flow inside the microchannel, as predicted by the simulation results shown in Figure 2c. The breadth of the punched holes (with respect to the width of the microchannel) was fixed, and the streamlines at different flow ratios of the inlets and outlets were simulated. The purpose of this simulation was to observe the height ratio of the sheath flow streamlines and particle mixture streamlines during steep focusing of the sample mixture streamlines in the perpendicular plane (see Figure S3a in the Supporting Information), and to study the consequences of the streamline height (at the outlets) on the separation efficiency (see Figure S3b in the Supporting Information).

## 3 Results and Discussion

### 3.1 Device Operation at Low Flow Rates

**Figure 3:**
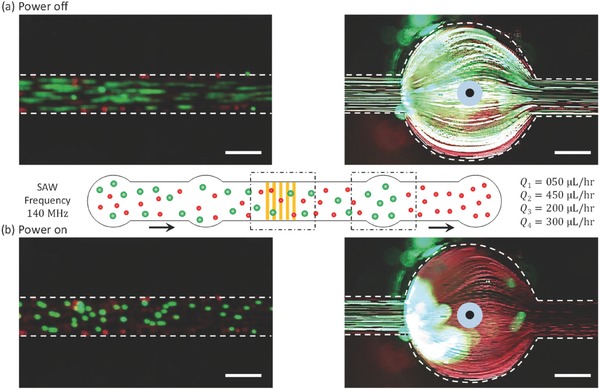
Photographic images of the particle separation experiment based on the upward movement of particles under an applied ARF. a) Power off: the particle mixture (green and red) flowed together through the lower streamlines and resulted in no separation. b) Power on: green particles migrated upward toward the upper streamlines, resulting in separation. Scale bar: 250 µm.

**Figure 4:**
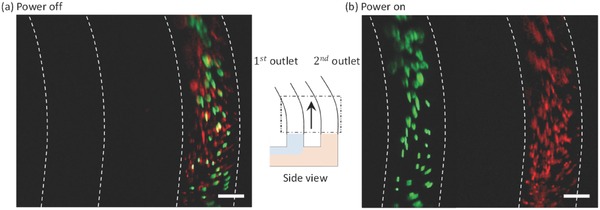
A side view graphic supporting the photographic images at the outlet pipes a) Power off: a control experiment showing that particles of different sizes were collected through the second outlet. b) Power on: larger (green) and smaller (red) particles were collected through the first outlet and second outlet, respectively. Scale bar: 250 µm.

A mixture of microspheres was separated using a single IDT placed under a straight microchannel, as shown in **Figure** **3**. Experimental images revealed the extent of particle separation under the applied vertical component of the ARF. The particle mixture (green, 4.8 µm and red, 2.0 µm) and DI water were injected through the first and second inlets with flow rates of 50 µL h^−1^ (*Q*_1_) and 450 µL h^−1^ (*Q*_2_), respectively. The first and second outlets were used to collect the particles at flow rates of 200 µL h^−1^ (*Q*_3_) and 300 µL h^−1^ (*Q*_4_), respectively. When the device was turned off, the large (green) and small (red) particles flowed together over the IDT along the lower streamlines below the sheath flow streamlines, as shown in Figure 3a. These conditions resulted in no separation of particles. For the acoustic waves with 140 MHz frequency propagating through the liquid medium, the κ‐factor values for the suspended 4.8 µm (green) and 2.0 µm (red) particles were calculated to be 1.42 and 0.59, respectively.^43^ When the device was actuated with an AC signal of 140 MHz frequency, the green particles were pushed upward due to the vertical component of the ARF (κ > 1), whereas the smaller red particles flowed along the lower streamlines (κ < 1), as shown in Figure 3b. The upward migration of the larger (green particles) may be observed in Video S1 in the Supporting Information. The green particles were collected at the first outlet whereas the red particles moved toward the second outlet. These effects produced the successful separation of particles, as indicated in the photographic images of the outlets, shown in **Figure** **4**.

A side view of the outlet pipes confirmed the separation of particles. When the power was turned off, all of the particles (green and red) were collected at the second outlet. Not a single particle flowed through the first outlet, as shown in Figure 4a. When the power was turned on, however, the particles were effectively separated as the larger particles (green) passed through the first outlet and the smaller (red) particles passed through the second outlet, as shown in Figure 4b. The separation of both particles (green and red) may be observed in the Videos S2 and S3 presented in the Supporting Information.

**Figure 5:**
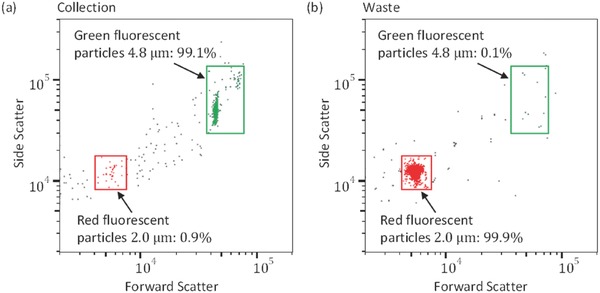
Particle separation results for *Q*_1_/*Q*_2_ = 1/9 and *Q*_3_/*Q*_4_ =  0.67/1. Flow cytometry graphs showing the ratio of particles of different sizes collected at the collection a) and waste b) outlets, respectively.

Flow cytometry was used to count the particles collected from the first outlet (collection) and the second outlet (waste) under the experimental conditions *Q*_1_/*Q*_2_ = 1/9 and *Q*_3_/*Q*_4_ = 0.67/1, as shown in **Figure** **5**. The size and location of the rectangle boxes were adjusted by using the flow cytometry results obtained from pure samples of green (large) and red (small) particles. Our aim was to isolate the green particles from the mixture of green and red particles by pushing the larger particles in the upward direction. The green particles were separated through the collection outlet with a 99.1% purity, and the red particles were separated through the waste outlet with a 99.9% recovery rate.

### 3.2 Characterization of Inlets and Outlets Flow Rate

**Figure 6:**
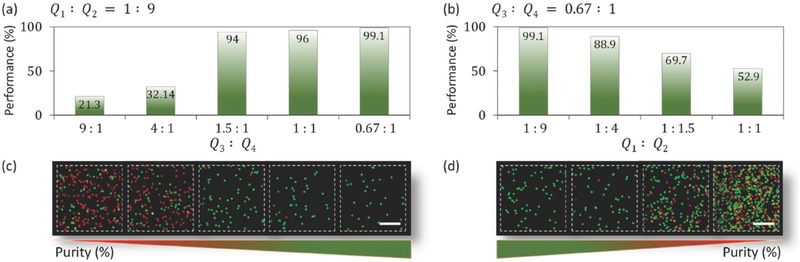
Particle separation purity measures at different ratios of the inlet and outlet flow rates. a) Percentage of purity at various outlet flow ratios *Q*_3_: *Q*_4_ was varied from 9:1 to 0.67:1, holding the inlet flow ratio fixed at *Q*_1_:  *Q*_2_ =  1: 9. b) Percentage of purities across inlet flow ratios of *Q*_1_: *Q*_2_ = 1: 9 to 1: 1, holding the outlet flow ratio fixed at *Q*_3_: *Q*_4_ = 0.67: 1. c,d) Hemocytometer images obtained showing the particle size compositions collected at different outlet and inlet flow ratios. Scale bar: 1 mm.

In addition to the κ‐factor, we examined the effect of the flow ratio at the inlets (*Q*_1_, *Q*_2_) and outlets (*Q*_3_, *Q*_4_). We first fixed the inlet flow ratio (*Q*_1_/*Q*_2_ = 1/9) so as to focus the particles in the lower streamlines while varying the flow rate ratio at the outlets. We started with an outlet flow ratio of *Q*_3_/*Q*_4_ = 9/1. Under these flow conditions, all of the green (large) particles were separated through the first outlet; however, a number of red (small) particles also made their way into the green particle stream, resulting in only a 21.3% separation efficiency. This low separation efficiency was attributed to the streaming flow produced by the TSAWs, which disturbed the laminar flow streamlines inside the microchannel and resulted in mixed particle sample collection at the outlets. The ratio of the sheath flow and sample flow heights above the IDT was adjusted to achieve a high separation efficiency. We decreased the height of the sheath flow and increased the height of the sample mixture flow after the IDT by increasing the flow rate *Q*_4_ and decreasing the flow rate *Q*_3_ until the point(*Q*_3_/*Q*_4_ =  0.67/1). Beyond this point, the green (large) particles passed through the second outlet along with the red particles, reducing the recovery rate. As shown in **Figure** **6**a, the separation efficiency increased as *Q*_3_ decreased compared with *Q*_4_. At *Q*_3_/*Q*_4_ =  1.5/1,  the efficiency reached 94% and exceeded 99% at *Q*_3_/*Q*_4_ =  0.67/1.

We studied the effect of the inlet flow ratio by fixing the outlet flow ratio. Disruptions to the flow separation by the streaming flow were avoided by selecting an outlet flow ratio at which the separation reached a maximum (*Q*_3_/*Q*_4_ =  0.67/1). The flow rate at the inlet was then varied to determine the effect of vertical particle focusing. As the inlets flow ratio *Q*_1_/*Q*_2_ was increased from 1: 9 to 1: 1, as shown in Figure 6b, the separation efficiency decreased because the sample mixture flow height increased and the sheath flow decreased. The particles were then dispersed under the sheath flow (not tightly focused). If the particles were dispersed prior to reaching the separation zone, a nonuniform ARF effect on the particles would have been observed. The flow ratios were adjusted accordingly to achieve a high separation efficiency. The particle samples collected from the outlets at different inlet and outlet flow rate conditions were quantitatively evaluated using the ImageJ software and a hemocytometer. Photographic images showing a top view of the hemocytometer are presented in Figure 6c,d for different flow ratios at the inlets and outlets.

**Figure 7:**
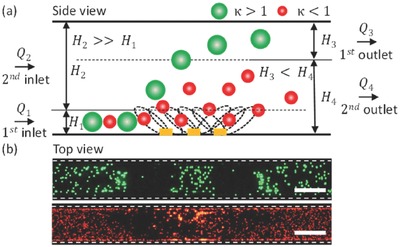
a) A side view schematic diagram illustrating the height of the sheath flow and the mixed particle flow within sections of the microchannel. Particles with κ > 1 were affected by the ARF, whereas particles with κ < 1 were predominantly influenced by the streaming flow. b) Top view of the experimental images, illustrating the effects of SAWs on particles of different sizes. Scale bar: 250 µm.

The experimental results obtained at *Q*_1_/*Q*_2_ =  1/9 and *Q*_3_/*Q*_4_ =  0.67/1 are interpreted as shown in the schematic diagram in **Figure** **7**a. As discussed earlier, the height of the sheath flow *H*_2_ before the separation zone should be sufficiently greater than *H*_1_(*H*_1_: *H*_2_ = 1: 9) to pinch the green and red particles in the lower streamlines. By contrast, after the separation zone, the height of the sheath flow streamlines *H*_3_ leaving through the first outlet should be smaller than the height of the streamlines *H*_4_ exiting through the second outlet (*H*_3_: *H*_4_ =  2: 3) to avoid the effect of acoustic streaming on the separation efficiency. Green particles with κ > 1 were affected by the ARF, whereas the red particles with κ < 1 were affected by the acoustic streaming. An experiment was performed to verify this prediction. Particles were held stationary inside the microchannel. When the power was turned on, the green particles with κ > 1 were pushed in the lateral and perpendicular directions due to the horizontal and vertical components of the ARF;^38^ however, red particles with κ < 1 were under the influence of the acoustic streaming flow,^38^ so they travelled along the circular streamlines, displaying mixing behavior inside the microchannel, as shown in Figure 7b. In addition, the diverse behaviors of both particles can be observed in Video S4 presented in the Supporting Information.

### 3.3 Device Operation at High Flow Rates

**Figure 8:**
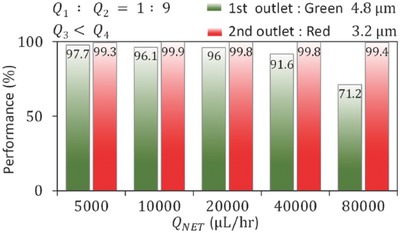
Purity and recovery measures at different net flow rates *Q*_NET_ by holding the conditions (*Q*_1_/*Q*_2_ =  1/9) and (*Q*_3_ < *Q*_4_) for the isolation of green, 4.8 µm, and red, 3.2 µm particles.

In addition to investigating the particles' separation using the principal component of ARF by uniquely focusing the particles in the vertical direction; we tested our device at higher flow rates for the separation of 4.8 and 3.2 µm particles. We fixed the inlet flow ratio (*Q*_1_/*Q*_2_ =  1/9) and passively regulated the outlet flow ratio (*Q*_3_/*Q*_4_) by differential fluidic resistance, and thus to reduce the number of external equipment. Flow rate at the first outlet *Q*_3_ should be greater than the flow rate at the second outlet *Q*_4_, as we already characterized by giving a negative pressure at one of the two outlets to avoid the effect of eckart streaming on the separation efficiency. **Figure** **8** shows the purity and recovery measures at the first and second outlets, respectively. Starting with the net flow rate, *Q*_NET_ = 5000 µL h^−1^ (cross‐sectional velocity: *V* = 69.4 mm s^−1^), we increased the *Q*_NET_ by doubling the prior one. As we increased the *Q*_NET_ from 5000 to 80 000 µL h^−1^ (*V* = 1110.4 mm s^−1^), the purity gradually decreases from 97.7% to 71.2%. In contrast, the recovery remains >99% even at a much higher flow rate (*Q*_NET_ = 80 000 µL h^−1^). Hemocytometer images of the sample collection at first and second outlets are presented in Figures S4 and S5 in the Supporting Information, respectively, for different net flow rates. The gradual decrease in the purity with the increase of *Q*_NET_ was investigated. The effect of streaming flow explained in Figure 7 might not play a major role in the decrease of purity at higher flow rates; therefore, we simulated the streamlines inside the microchannel to see the behavior of streamlines at higher flow rates by varying the port size (inlets, outlets) and net flow rate (*Q*_NET_).

Streamlines were simulated inside 250 µm wide microchannel with 1 mm inlets and outlets port size, holding the inlets and outlets flow ratio constant (*Q*_1_/*Q*_2_ =  1/9; *Q*_3_/*Q*_4_ =  0.67/1) as characterized before. As the *Q*_NET_ increased from 500 to 40 000µL h^−1^, there was a formation of vortices at the first outlet due to the sudden change in the geometry. Although *Q*_3_ < *Q*_4_ a few sample flow streamlines were still going with the sheath flow streamlines resulting in a decrease of purity at the first outlet (see Figure S6 in the Supporting Information). This effect of vortices formation can be minimized by decreasing the port size. Simulation of streamlines inside the microchannel with 500 µm diameter ports shows that there is a decrease of vortices formation at the first outlet (see Figure S7 in the Supporting Information). This problem can further be minimized by using the hole size equal to the width of the microchannel in order to achieve high purity at both outlets.

### 3.4 Tape‐Based Microchannel for the Separation of Particles

It should be noted that a simple, straight, manually fabricated tape‐based microchannel was used to successfully separate particles using the horizontal and vertical components of the ARF. A mixture of 4.8 µm (green) and 2.0 µm (red) particles, at the characterized flow rate conditions (*Q*_1_/*Q*_2_ =  1/9; *Q*_3_/*Q*_4_ =  0.67/1), was used to demonstrate the separation process. Figure S8a in the Supporting Information shows the separation of particles inside the microchannel, supporting with the experimental images of side view of the outlet pipes (see Figure S8b in the Supporting Information).

## 4 Conclusion

We demonstrated the use of vertical hydrodynamic focusing to separate microparticles in a straight microchannel using a single sheath flow. This technique is dissimilar from the previously reported methods of particle focusing in the SAW‐based separation devices that used a pair of sheath flows which horizontally focused particles in the center of a microchannel. The vertical focusing mechanism employed here was implemented using a smaller device footprint compared with that used in horizontal focusing techniques. Unlike previous acoustofluidic particle separation techniques, we used the vertical component of the ARF to separate particles in a straight single‐layered microchannel. The SAWs that leaked into water exerted a force along the vertical direction (*F*_v_) that far exceeded the force exerted in the horizontal direction (*F*_h_), i.e., *F*_v_ ≅ 2.5 *F*_h_. After hydrodynamically pinching the particles in the downward streamlines (vertical direction), the horizontal component of the ARF was used to slow down the motions of the larger particles while at the same time pushing them upward using the vertical (major) component of the ARF. We took advantage of the major component of the ARF to continuously separate 4.8 μm (green) from 2.0 μm (red) and 3.2 μm (red) polystyrene microparticles. This method enables the highly efficient (>99% purity and recovery rates) separation of particles over a wide range of flow rates using tens of milliwatts of power. Moreover, the device operates at higher flow rates (up to 80 000 µL h^−1^) with reasonable separation performance (purity 71.2% and recovery 99.4%). Furthermore, we fabricated a straight microchannel using a strip of tape manually cut into the desired shape. This method costs less than $1 for microchannel fabrication. This microchannel fabrication procedure can replace standard micro‐electro‐mechanical systems (MEMS) and soft‐lithography device fabrication processes that cost more than $500 due to the use of expensive glass/chrome‐based photomasks. These characteristics render this particle separation technique a useful addition to micro total analysis systems and point‐of‐care devices.

## 5 Experimental Section

*Device Fabrication*: The acoustofluidic device as shown in Figure 2a was composed of a piezoelectric (Lithium Niobate, LiNbO_3_) substrate (500 µm thick, 128° *Y*‐*X* cut, MTI Korea, Korea) with an IDT deposited on the top and a PDMS microfluidic channel. A bimetallic (Cr/Au, 300 Å/1000 Å) layer of interdigitated electrodes was deposited, using the e‐beam evaporation method and lift‐off process, to form an IDT having a total aperture of 1 mm and 20 electrode finger pairs with uniform widths and spacings in between (λ/4 = 6.5 μm). A thin layer of SiO_2_ (2000 Å) was also deposited on top of the electrodes using plasma‐enhanced chemical vapor deposition to keep them safe from mechanical damage and enhance bonding.

A commonly used soft photolithographic process was used to fabricate PDMS microchannel. A PDMS base and a curing agent (Sylgard 184A and 185B, Dow corning, USA) were mixed in a 10:1 ratio and poured on the top of the SU‐8 mold. PDMS was cured at 65 °C for at least 2 h and later on peeled off the surface‐treated silanized Si substrate. After the inlet and outlet ports were punched through the PDMS using punching tool (Harris Uni‐Core), the microchannel and LiNbO_3_ substrate were exposed to oxygen plasma^51^ in a multipurpose oxygen plasma system (Covance, Femto Science, Korea) for 2 min at 150 W and 750 mTorr. The microchannel was bonded to the substrate in such a way that IDT would be manually placed between the second inlet and first outlet. As the PDMS microchannel was 250 µm in width and 80 µm in height, it did not require a tight alignment against the IDT with 1 mm aperture. In addition to soft photolithographic process, microchannel was also fabricated manually using a strip of tape. Detail regarding the tape‐based microchannel fabrication and experimental setup can be found in the Supporting Information.

*Particles Solution Preparation*: Three different‐sized polystyrene particles having the same density were used in two pairs (4.8 + 2.0 µm, and 4.8 + 3.2 µm) to demonstrate the separation mechanism: 4.8 µm green fluorescent (<5% uniformity, No. G1000, Thermo Scientific, CA, USA), 3.2 µm red fluorescent (<5% uniformity, No. R0300, Thermo Scientific, CA, USA), and 2.0 µm red fluorescent (<5% uniformity, No. R0200, Thermo Scientific, CA, USA). Each of the particle solution consisted of 1% solid microspheres per microliter of liquid as the total number of beads/µL was counted (green 4.8 µm: 1.72 × 10^5^, red 3.2 µm: 5.81 × 10^5^, red 2.0 µm: 2.39 × 10^6^) for each particle solution. To add an equal number of particles in a sample, 13.9 µL of green (4.8 µm) and 1 or 4.11 µL of red (2.0 or 3.2 µm) particle solutions per 1 mL of DI water were added. Later on, 21.8 wt% glycerin and 1 wt% tween 20 were added.^52^ The addition of glycerin matched the density of the particles with water and prevented the particles from settling down in water. Tween 20 was added to avoid the formation of doublets and triplets of the particles by preventing particle‐to‐particle adhesion.

## Conflict of Interest

The authors declare no conflict of interest.

## Supporting information

Supplementary

Supplementary

Supplementary

Supplementary

Supplementary
