# Supplementary material for: Vertical Hydrodynamic Focusing and Continuous Acoustofluidic Separation of Particles via Upward Migration
Source: Adv Sci (Weinh). 2017 Dec 22;5(2):1700285. doi: 10.1002/advs.201700285 (PMC5827645; doi:10.1002/advs.201700285)
Supplement: Supplementary file 1 — Supplementary [file ADVS-5-1700285-s002.pdf]

Copyright WILEY-VCH Verlag GmbH & Co. KGaA, 69469 Weinheim, Germany, 2017.

ADVANCED SCIENCE
Open Access

Supporting Information

for *Adv. Sci.,* DOI: 10.1002/advs.201700285

Vertical Hydrodynamic Focusing and Continuous Acoustofluidic Separation of Particles via Upward Migration

*Husnain Ahmed, Ghulam Destgeer, Jinsoo Park, Jin Ho Jung, and Hyung Jin Sung**

Copyright WILEY-VCH Verlag GmbH & Co. KGaA, 69469 Weinheim, Germany, 2017.

Supporting Information

Vertical Hydrodynamic Focusing and Continuous Acoustofluidic Separation of Particles via Upward Migration

*Husnain Ahmed, Ghulam Destgeer, Jinsoo Park, Jin Ho Jung, and Hyung Jin Sung**

1. Tape based microchannel fabrication

In addition to standard MEMS and soft lithography method of microchannel fabrication, we also fabricated the microchannel using scotch tape manually; as height and width of the microchannel does not play a critical role in our device for particle separation. In contrast to spin coating a photoresist on top of a silicone substrate and then making a mold of a photoresist using photomask and UV aligner, a piece of a polyimide silicone adhesive tape (thickness of 70 µm that defines microchannel height, Tianjin Fortune, China) was manually cut (width ~800 µm) and pasted on top of the Si substrate which acted as a mold for the PDMS microchannel. After a mixture of PDMS and its curing agent (10:1) was poured on top of the scotch-tape mold, it was vacuumed to remove unnecessary bubbles, and was baked at 65 °C for at least two hours. Rest of the process is similar to what has already been explained in the previous section. This process of making a microchannel could be a cost-effective alternative compared to the standard soft lithography process.

2. Experimental Setup

The device was mounted on top of a fluorescent microscope stage (BX53, Olympus, Japan) for visualization using an appropriate lens (2x, 4x or 10x magnification). The mixture of fluorescent polystyrene particles with diameters 4.8 µm (green) and 2.0 µm (red) was pumped (neMESYS, Cetoni GmbH, Germany) into the microchannel through the first inlet. DI water

was pumped as a sheath flow through the second inlet to force the particles solution to flow in the lower streamlines. One of the two outlets was connected with a pump to suck the fluid out by applying a negative pressure and another outlet was opened to atmospheric pressure for the collection of sample for analysis. The inlet sample to sheath flow ratio was 1:9. The flow rates at the outlets were readily adjusted to achieve a maximum separation efficiency. The net flow rate was varied from 500 µL/hr to 80,000 µL/hr. An RF signal generator (N5181A, Agilent Technologies, U.S.A) was used to generate an AC signal at 140 MHz frequency and tunable amplitude 4.5-50 mV (dependent on net flow rate) that was amplified (LZT-22+, Mini-Circuits, U.S.A) up to 11-1330 mW before feeding into the device. The IDTs were deposited parallel to the primary axis of the lithium niobate wafer such that the speed of sound in the direction perpendicular to the IDTs was ~3650 m/s, while the actuation frequency was estimated to be $f \cong$ 3650 ms^{-1} / 26 µm $\cong$ 140 MHz. The images of green and red fluorescent particles were captured by CCD camera (DP72, Olympus, Japan) using separate filters and later on stacked together to obtain a single image. The captured microscopic images were processed to remove the background noise using ImageJ (http://imagej.nih.gov/ij/) software for better image quality. The collected sample particles at two outlets were analyzed through ImageJ software and counter-analyzed with a c-chip disposable hemocytometer (Digital Bio, Korea). At the end, the particles were counted using flow cytometry at the specific flow ratios at the inlets and outlets to confirm the efficient separation of the particles.

3. List of graphics

Supporting video 1.

The upward migration of the larger green (4.8 µm) particles.

Supporting video 2.

Particle separation shown at the first outlet.

Supporting video 3

Particle separation shown by the side view of the outlet pipes.

Supporting video 4

The diverse behaviors of both particles (green, 4.8 µm ; red, 2.0 µm)

Figure S1.

SAW-based particle separation using horizontal and vertical component of acoustic radiation force (ARF).

Figure S2.

Simulation of streamlines inside the microchannel at different inlets and outlets port size.

Figure S3.

Simulation of streamlines inside the microchannel at different inlets and outlets flow ratio.

Figure S4.

Hemocytometer images of sample collection at first outlet for device operation at high flowrates.

Figure S5.

Hemocytometer images of sample collection at second outlet for device operation at high flowrates.

Figure S6.

Simulation of the streamlines inside 250 µm wide microchannel with 1mm punched hole.

Figure S7.

Simulation of the streamlines inside 250 µm wide microchannel with 500 µm punched hole.

Figure S8.

Experimental images of particle separation inside a manually fabricated tape-based microchannel.

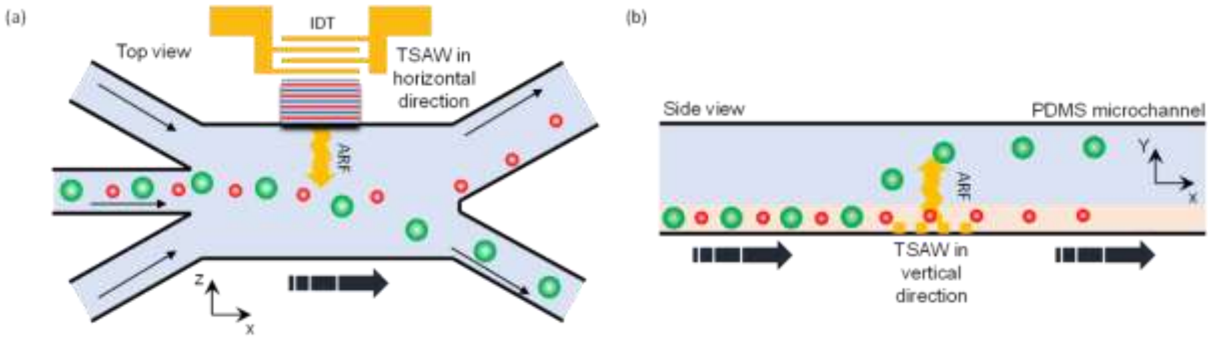

Figure S1. SAW-based particle separation using horizontal (a) and vertical (b) component of acoustic radiation force (ARF).

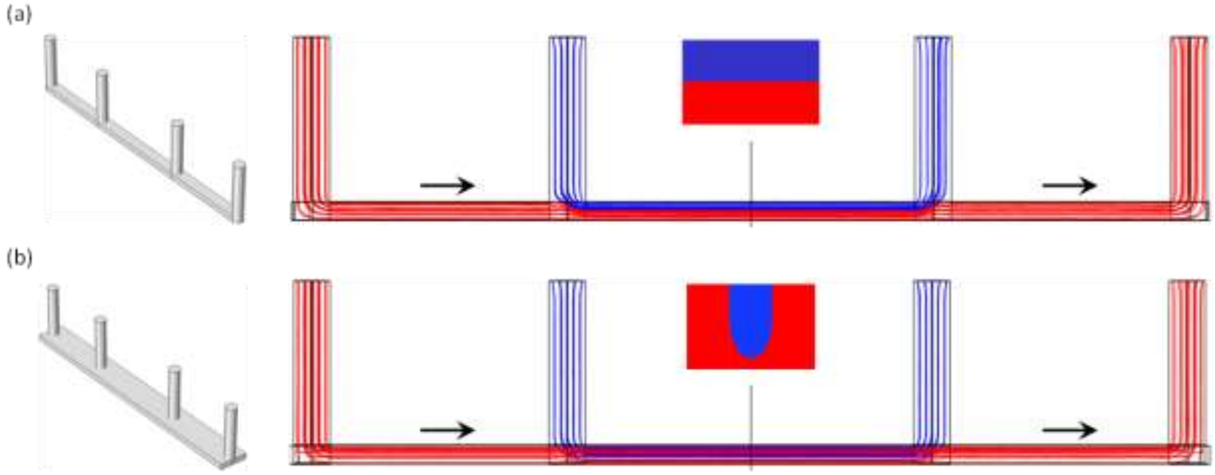

Figure S2. (a) A 3D geometry and side view of the simulation of streamlines inside the straight microchannel when the diameter of the punched hole is smaller than the width of the microchannel. (b) The diameter of the punched holes is equal to the width of the microchannel.

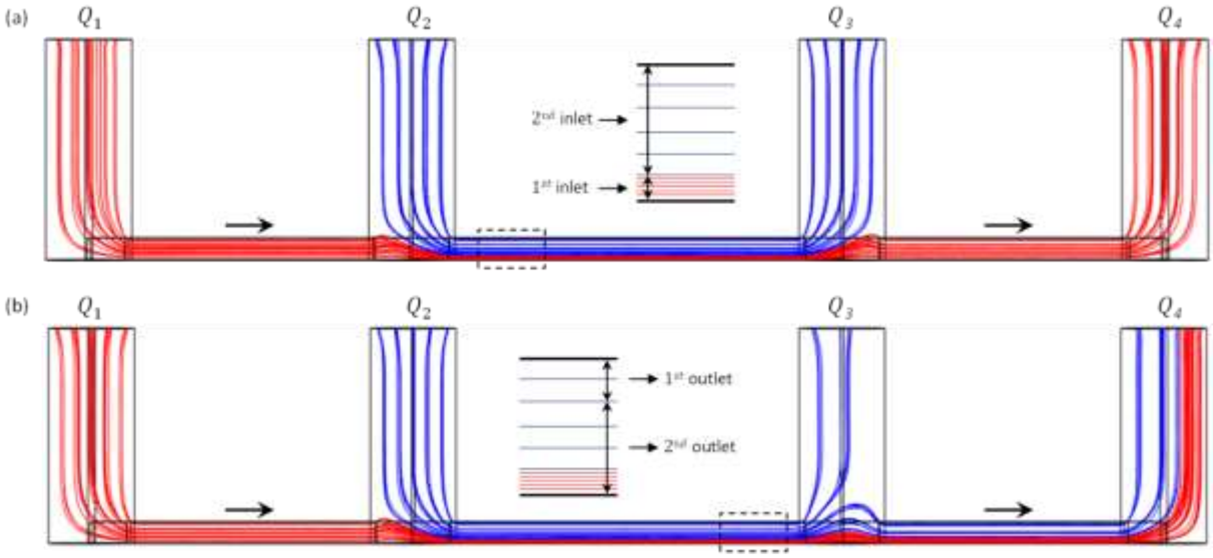

Figure S3. (a) Simulation of the streamlines inside the microchannel at $Q_1/Q_2 = Q_4/Q_3$ with zoom in side view of the streamlines after the 1st inlet. (b) Simulation of the streamlines inside the microchannel at $Q_1/Q_2 < Q_4/Q_3$ with zoom in side view of the streamlines leaving through 1st and 2nd outlet.

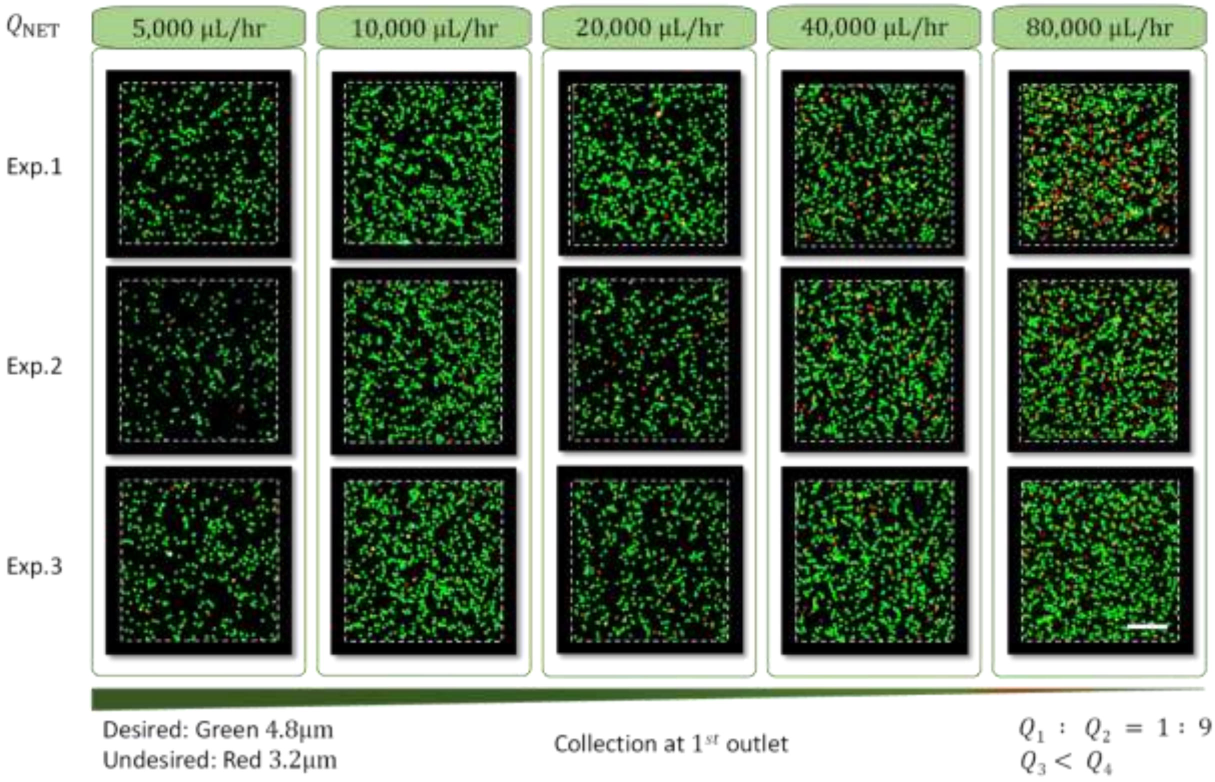

Figure S4. Hemocytometer images of sample collection at first outlet. Varied the net flowrates (Q_{NET}) by fixing the inlets and outlets flow ratio, $Q_1 : Q_2 = 1:9$ and $Q_3 < Q_4$ for the separation of green, 4.8 µm and red, 3.2 µm particles. Scale bar: 1 mm.

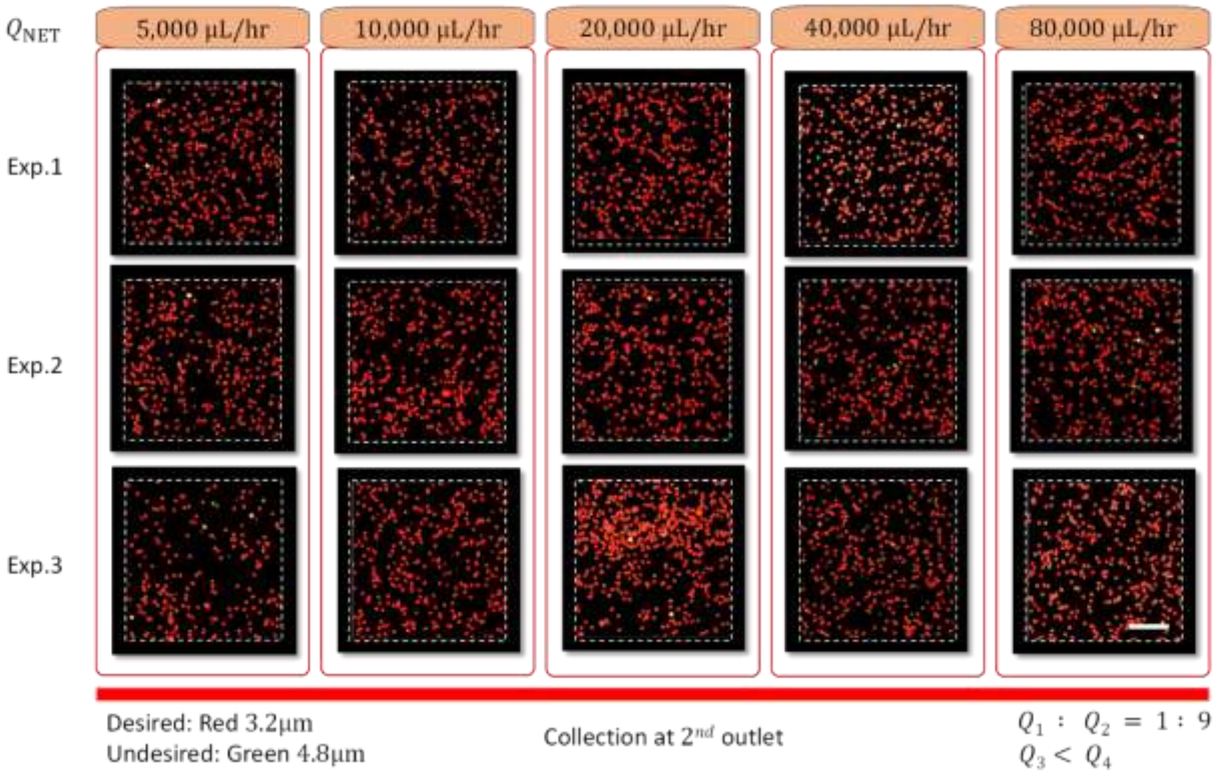

Figure S5. Hemocytometer images of sample collection at second outlet. Varied the net flowrates (Q_{NET}) by fixing the inlets and outlets flow ratio, $Q_1 : Q_2 = 1:9$ and $Q_3 < Q_4$ for the separation of green, 4.8 µm and red, 3.2 µm particles. Scale bar: 1 mm.

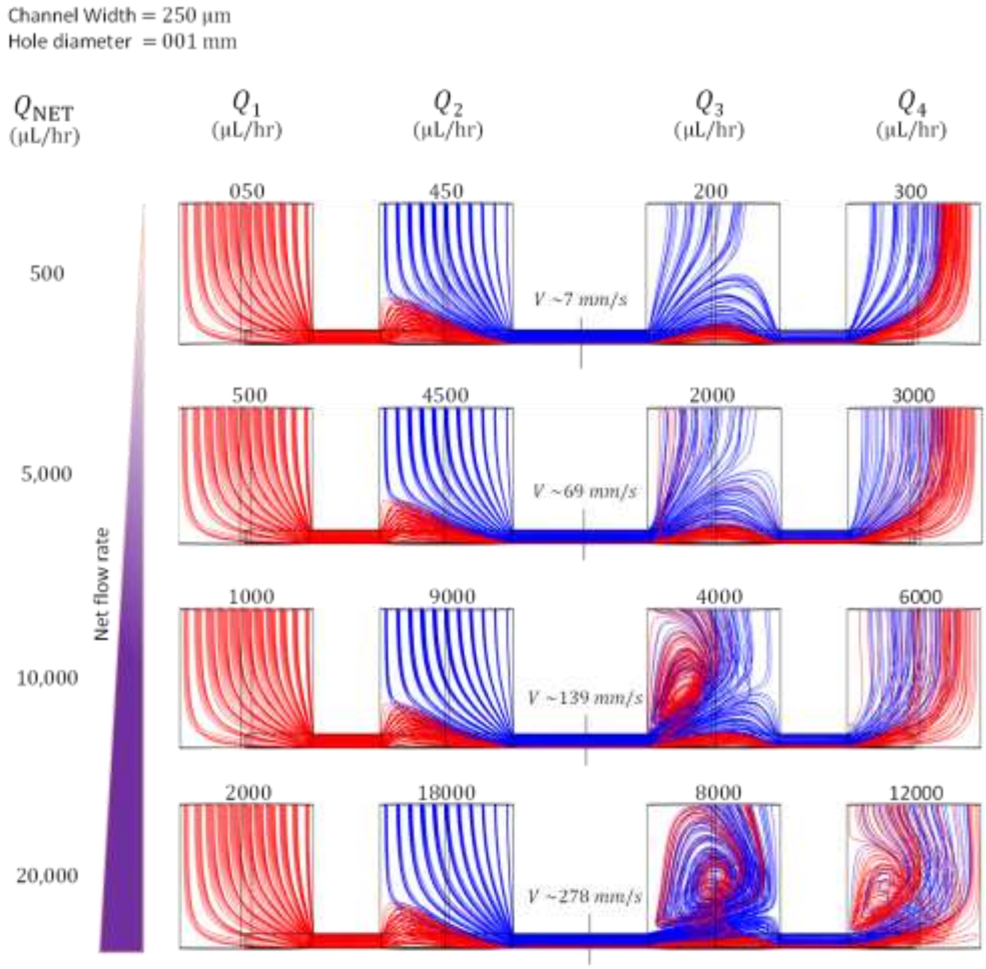

Figure S6. Simulation of the streamlines inside 250 µm wide microchannel with 1mm punched hole at $Q_1/Q_2 = 1/9$ and $Q_3/Q_4 = 0.67/1$ for different net flowrates.

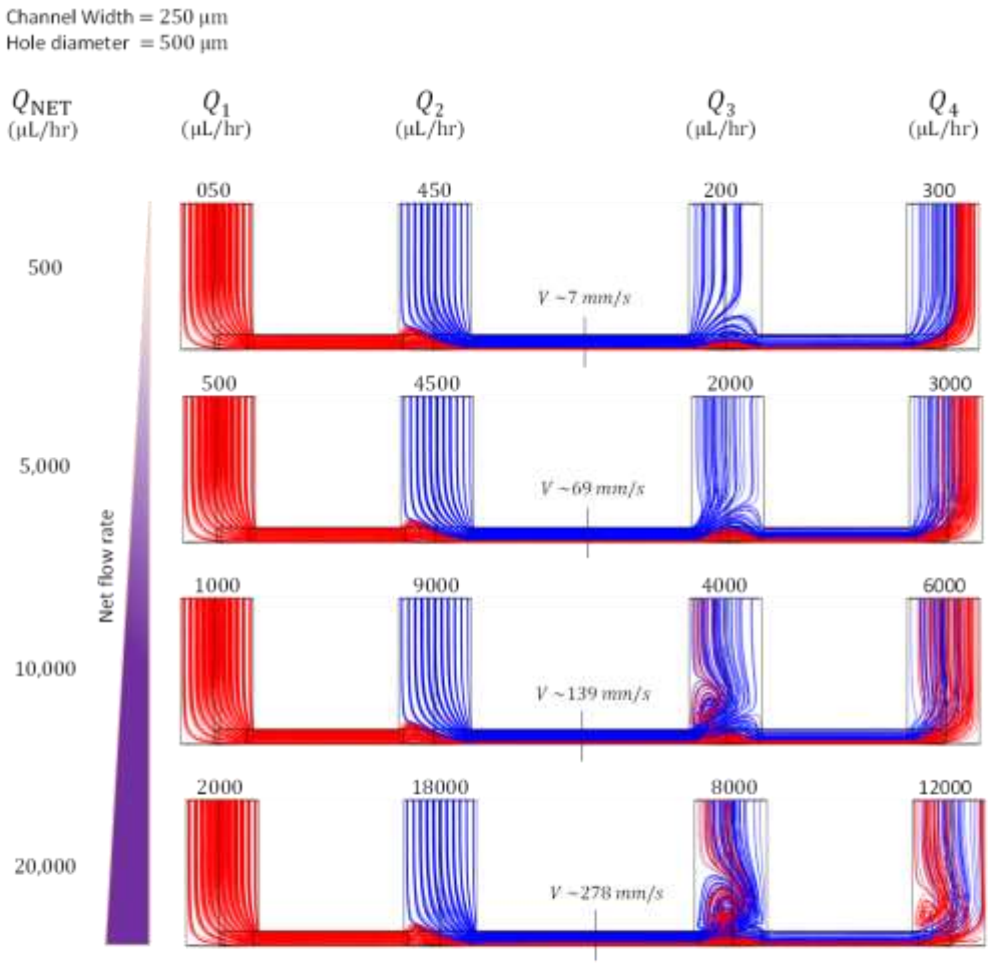

Figure S7. Simulation of the streamlines inside 250 µm wide microchannel with 500 µm punched hole at $Q_1/Q_2 = 1/9$ and $Q_3/Q_4 = 0.67/1$ for different net flowrates.

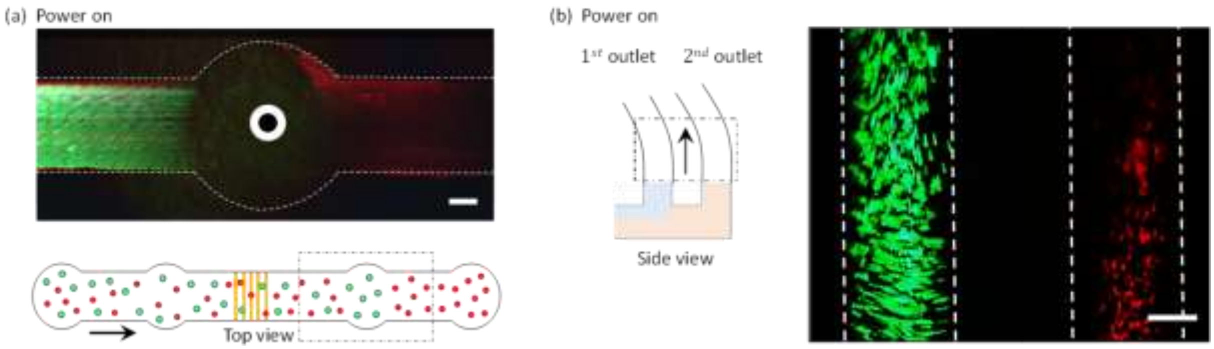

Figure S8. (a) A top view of the schematic with an experimental image of the manually fabricated tape based microchannel and (b) side view diagram of the outlet pipes with its photographic image. Scale bar: 250 µm.
